# A Single-Cycle Influenza A Virus-Based SARS-CoV-2 Vaccine Elicits Potent Immune Responses in a Mouse Model

**DOI:** 10.3390/vaccines9080850

**Published:** 2021-08-03

**Authors:** Surapong Koonpaew, Challika Kaewborisuth, Kanjana Srisutthisamphan, Asawin Wanitchang, Theeradej Thaweerattanasinp, Janya Saenboonrueng, Sukontip Poonsuk, Juggragarn Jengarn, Ratchanont Viriyakitkosol, Jarin Kramyu, Anan Jongkaewwattana

**Affiliations:** 1 Virology and Cell Technology Research Team, National Center for Genetic Engineering and Biotechnology (BIOTEC), National Science and Technology Development Agency (NSTDA), Pathumthani 12120, Thailand; Surapong.koo@biotec.or.th (S.K.); Challika.kae@biotec.or.th (C.K.); Kanjana.sri@biotec.or.th (K.S.); Asawin.wan@biotec.or.th (A.W.); theeradej.tha@biotec.or.th (T.T.); Janya@biotec.or.th (J.S.); Sukontip.poo@biotec.or.th (S.P.); Juggragarn.jen@biotec.or.th (J.J.); Jarin@biotec.or.th (J.K.); 2 Faculty of Veterinary Science, Chulalongkorn University, Bangkok 10330, Thailand; ratchanont.v@student.chula.ac.th

**Keywords:** SARS-CoV-2, single-cycle influenza virus-based vaccine, spike RBD, spike-pseudotyped virus

## Abstract

The use of virus-vectored platforms has increasingly gained attention in vaccine development as a means for delivering antigenic genes of interest into target hosts. Here, we describe a single-cycle influenza virus-based SARS-CoV-2 vaccine designated as scPR8-RBD-M2. The vaccine utilizes the chimeric gene encoding 2A peptide-based bicistronic protein cassette of the SARS-CoV-2 receptor-binding domain (RBD) and influenza matrix 2 (M2) protein. The C-terminus of the RBD was designed to link with the cytoplasmic domain of the influenza virus hemagglutinin (HA) to anchor the RBD on the surface of producing cells and virus envelope. The chimeric RBD-M2 gene was incorporated in place of the HA open-reading frame (ORF) between the 3′ and 5′ UTR of HA gene for the virus rescue in MDCK cells stably expressing HA. The virus was also constructed with the disrupted M2 ORF in segment seven to ensure that M2 from the RBD-M2 was utilized. The chimeric gene was intact and strongly expressed in infected cells upon several passages, suggesting that the antigen was stably maintained in the vaccine candidate. Mice inoculated with scPR8-RBD-M2 via two alternative prime-boost regimens (intranasal-intranasal or intranasal-intramuscular routes) elicited robust mucosal and systemic humoral immune responses and cell-mediated immunity. Notably, we demonstrated that immunized mouse sera exhibited neutralizing activity against pseudotyped viruses bearing SARS-CoV-2 spikes from various variants, albeit with varying potency. Our study warrants further development of a replication-deficient influenza virus as a promising SARS-CoV-2 vaccine candidate.

## 1. Introduction

Severe acute respiratory syndrome coronavirus (SARS-CoV-2), a newly emerged human coronavirus, has been identified as a causative agent of COVID-19 and has led to a global pandemic. Effective vaccines that elicit efficient adaptive immunity, including B and T cell responses against SARS-CoV-2, are essential for the fight against the spread of the virus and the return to pre-COVID-19 normalcy. While several COVID-19 vaccines [1,2] have been manufactured and used successfully to control the outbreaks in many countries, the need for greater and quicker access is critical against a backdrop of emerging variants, especially in developing countries and with vulnerable populations. The influenza viral vector systems offer one of the most flexible vaccine platforms, which can be rapidly developed to uphold efficacy against both original and new emerging variants.

Well-established reverse genetics techniques have shown to facilitate rapid influenza virus recovery [3]. Moreover, these methods have been successfully applied for expressing foreign proteins or antigenic regions with great potentials for vaccine development. Influenza A virus (IAV) vectors harboring foreign antigens have proven successful in inducing humoral immune responses against pathogen-derived proteins, including West Nile virus, Bacillus anthracis, HIV, and botulinum neurotoxin in several preclinical animal models [4,5,6,7]. These studies demonstrated that the insertion of foreign genes into IAV gene segments encoding viral surface proteins, such as hemagglutinin (HA) or neuraminidase (NA), could provide protective immunity against relevant antigens. The genes or epitopes of interest could be cloned into either HA/NA antigenic sites, in frame with the 5′ end of the viral gene, or replacing the majority of the open reading frames (ORFs). Similar to SARS-CoV-2, the influenza A virus is a major human respiratory virus that could transmit to a new host via mucosal membranes of the respiratory and gastrointestinal epithelial linings. Accordingly, IAV-based vector vaccines developed against various pathogens could be administered via several routes, including intramuscularly and intranasally to broaden their utilization in inducing protective immunity.

A single-cycle influenza A virus (scIAV)-based vaccine was designed to allow the expression of foreign genes and served as a safe viral-vectored vaccine candidate [8] for COVID-19. The SARS-CoV-2 S protein is made up of two distinct subunits, S1 and S2. The receptor-binding domain (RBD) localized within the S1 subunit is known to bind the angiotensin-converting enzyme 2 (ACE2) receptor on the host cell surface, leading to the fusion of the viral and cellular membranes mediated by the S2 subunit [9]. The SARS-CoV-2 spike RBD is known to contain neutralizing epitopes and multiple dominant T cell epitopes [10]. As a result, SARS-CoV-2 RBD-specific immunities could confer robust protective immunity against SARS-CoV-2.

In this study, we aimed to generate a scIAV-based SARS-CoV-2 vaccine to improve its safety and immunogenicity. The prototype influenza A/Puerto Rico/8/1934 (H1N1, PR8) strain was chosen as the viral backbone for the generation of a scIAV-based vaccine (scPR8) due to its attenuated phenotype in humans [11]. The synthetic gene encoding the SARS-CoV-2 receptor-binding domain (RBD) fused with the cytoplasmic domain of IAV HA was inserted in the coding region of the PR8 HA gene to generate a membrane-anchored RBD on the viral envelope. The chimeric gene was also engineered to improve the inserted gene segments’ stability and biochemical characteristics by placing 2A-self cleavage signal peptide to co-translate the RBD and IAV M2 protein (RBD-M2). Due to the lack of intrinsic HA protein, the scPR8-RBD-M2 requires HA-expressing host cells to provide HA in trans for virus propagation. Vaccine immunogenicity testing was performed in BALB/c mouse model under distinct prime-boost regimens. The presence of RBD-specific IgG in serum and IgA in lung supernatants as well as a cell-mediated immune response in spleen-derived mononuclear cell samples were measured after boosting. In addition, immunized mice serum neutralization values were investigated against pseudotyped viruses displaying different spike variants. Given the ease of SARS-CoV-2 gene modification and the rapidity of recombinant influenza virus production via reverse genetics, the influenza virus-vectored vaccine platform could become another alternative for combating SARS-CoV-2.

## 2. Materials and Methods

### 2.1. Cell Lines

Human embryonic kidney (HEK293T; ATCC CRL-11268) and Madin–Darby canine kidney (MDCK; ATCC CCL-34) cells were maintained in Opti-MEM^®^ medium (Gibco^TM^, Invitrogen, Carlsbad, CA, USA) supplemented with 10% fetal bovine serum (FBS) and 1X antibiotic-antimycotic (100 untis/mL of penicillin, 100 µg/mL of streptomycin and 0.25 µg/mL of Gibco Amphotericin B; Gibco^TM^). Cells were grown at 37 °C in a 5% CO_2_ atmosphere.

MDCK cells stably expressing the HA protein (MDCK-HA) derived from A/Puerto Rico/8/1934 (H1N1, PR8) have been previously described [12]. Briefly, the full-length HA gene was PCR amplified from pHW2000-PR8 HA and subsequently inserted into the pSIN-CSGW-UbEm under the spleen focus-forming virus (SFFV) promoters (pUb-HA plasmid) [13]. HEK293T cells were co-transfected with pUb-HA plasmid, a packaging plasmid (pCMV-ΔR8.91), and an envelope expression plasmid (pMD2.VSVG) for the recombinant lentivirus rescue. Cell supernatants containing the lentivirus were collected at 48 h post transfection (hpt) and adsorbed onto MDCK cells. A single clone of transduced MDCK cell expressing HA was selected, and the HA protein expression was confirmed by Western blot analysis. The MDCK-HA clone that gave rise to the highest hemagglutination (HA) titer of the HA-deficient IAV was chosen and used in further experiments.

### 2.2. Plasmid Construction

The codon-optimized construct, tPA-RBD-HAcyt-2A-M2 (RBD-M2) comprising the signal peptide of human tissue plasminogen activator (tPA), the RBD of SARS-CoV-2 spike (Wuhan-Hu-1 (EPI_ISL_402125); amino acid 325–532), the transmembrane and the cytoplasmic tail of the PR8 HA (amino acid 517–565), -2A and the PR8 M2 was synthesized for high expression in human cells (Genscript, Piscataway NJ, USA). To generate the plasmid expressing RBD-M2 for IAV reverse genetics, the pHW2000-PR8 HA plasmid was used as a template to remove the original HA, leaving the 3′ non-coding region (NCR) and 100 nucleotides (nt) of 3′ HA, and the 5′ NCR, and 180 nt of 5′ HA of linearized plasmid (pHW-ΔHA-PS). The pHW-ΔHA-PS was subsequently ligated with the RBD-M2 construct by In-Fusion™ cloning technique (Takara Bio, Mountain View, CA, USA). In addition, the reporter gene, mCherry, was also cloned into the pHW-ΔHA-PS for making the pHW-ΔHA-mCherry as a plasmid control.

pHW-ΔM2 plasmid was constructed as described previously [12]. Briefly, pHW2000-M (PR8) was subjected to site-directed mutagenesis to introduce two consecutive stop codons (W41Stop, I42Stop). Moreover, to prevent the expression of functional M2, M42 [14], an additional nucleotide was introduced after the stop codon of the M1 gene to disrupt the M2 reading frame. All plasmids were subject to direct nucleotide sequencing (First Base, Selangor, Malaysia).

SARS-CoV-2 spikes (Wuhan-Hu-1, B.1.1.7 (Alpha; EPI_ISL_601443), B.1.351 (Beta; EPI_ISL_700428), and P.1 (Gamma; EPI_ISL_984620)) were codon-optimized and synthesized (Genscript). Each synthetic gene was cloned into pCAGGS expression plasmid. Sequences used for gene synthesis were derived from the GISAID database.

### 2.3. Rescue of a Single-Cycle IAV-Based SARS-CoV-2 Virus (scPR8-RBD-M2)

**Figure 1:**
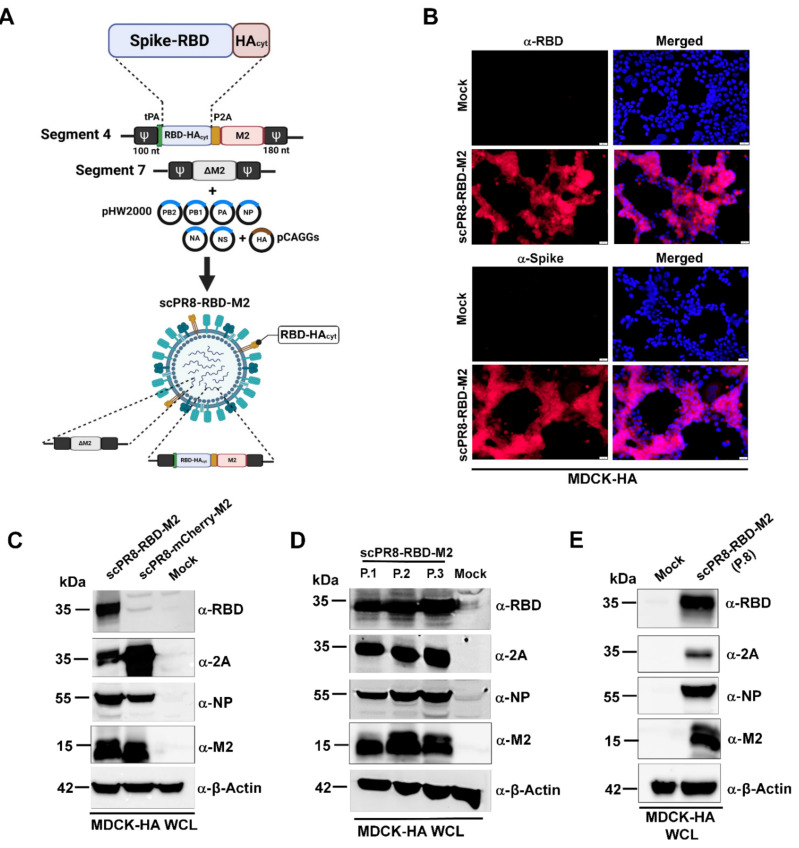
Generation and characterization of scPR8 containing receptor binding domain (RBD) of SARS-CoV-2 spike protein (scPR8-RBD-M2). (**A**) Schematic diagram of a reverse genetics-generated scPR8-RBD-M2. The chimeric gene cassette comprising tPA followed by a bicistronic RBD and M2 protein expression was introduced in the segment 4 (HA). The packaging signal sequence (Ψ) at both 3′ and 5′ terminal ends are also depicted. (**B**). scPR8-RBD-M2 infected MDCK-HA cells were assessed for RBD-HAcyt expression by (B)IFA using α-RBD and α-spike antibodies (scale bar indicates 100 μm) and (**C**) Western blot using α-RBD, -2A, -NP and –β actin as primary antibodies. Lower band(s) in lane 2 detected by α-2A antibody possibly indicate partially degraded mCherry-2A protein. (**D**) Stability of RBD-M2 inserted in infected MDCK-HA cells after several passages and (**E**) at the 8th passage was determined by Western blot. Mock infected MDCK-HA cells were used as a control. WCL: whole cell lysate.

scPR8-RBD-M2 viruses were recovered using the 9-plasmid reverse genetics rescue system as described previously [15]. Briefly, HEK293T cells were transfected with pHW2000 containing PR8 genes (PB2, PB1, PA, NP, NA, M (ΔM2) and NS) and RBD-M2 together with pCAGGS expressing PR8 HA (Figure 1A). A control virus expressing mCherry (scPR8-mCherry-M2) was generated by replacing RBD-M2 with the mCherry reporter gene in the PR8 HA segment. The viruses were propagated and serially passaged in MDCK cells stably expressing PR8 (MDCK-HA). Viral RNA was extracted using Viral Nucleic Acid Extraction Kit II (Geneaid, New Taipei City, China). According to the manufacturer’s guidelines, reverse transcriptase polymerase chain reaction (RT-PCR) was performed using PrimeScript™ OneStep RT-PCR Kit Ver.2 (Takara Bio Mountain View, CA, USA,). Primers used were Bm-HA-1: 5′-TATTCGTCTCAGGGAGCAAAAGCAGGGG-3′, Bm-NS-890R: 5′-ATATCGTCTCGTATTAGTAGAAACAAGGGTGTTTT-3′ [16]. Infected MDCK-HA cells were collected and subjected to Western blot analysis to confirm whether the inserted RBD-M2 in the HA segment was maintained in the scPR8-RBD-M2 genome.

### 2.4. Growth Curve Analysis and Virus Titration

To assess the growth of scPR8-RBD-M2 compared to the wild-type (WT) PR8, MDCK and MDCK-HA cells were infected with the viruses at an MOI of 0.01 and maintained in Opti-MEM^®^ containing 2 µg/mL of TPCK-treated trypsin at 37 °C. Cell supernatants were collected at indicated time points. For virus titration, MDCK-HA cells grown in flat-bottom 96-well plates were inoculated with serial 10-fold dilutions of scPR8-RBD-M2 and incubated at 37 °C for 72 h. scPR8-RBD-M2-infected MDCK-HA cells were observed microscopically with fluorescent conjugated secondary antibodies recognizing monoclonal antibodies (MAb) against influenza virus NP (FluA-NP 4F1) (Southern Biotech, Birmingham, AL, USA). Virus titers were calculated by Reed and Muench method (TCID_50_/_mL_) [17].

### 2.5. Confocal Microscopy

MDCK-HA cells were grown on coverslips in 6-well plates and infected with scPR8-RBD-M2 and scPR8-mCherry-M2 viruses for 48 h. After infection, cells were fixed with 4% paraformaldehyde at 4 °C for 20 min and blocked under non-permeabilized and permeabilized conditions with 5% FBS and 0.5% BSA at room temperature for 1 h. Cells were then probed with rabbit anti-RBD or anti-S antibodies (Sino Biological, Beijing, China). Goat anti-rabbit antibodies conjugated with Alexa Fluor^®^ 488 (Abcam, Cambridge, UK) were used as secondary antibodies. After washing, cells were mounted with Prolong^TM^ Gold Antifade Mountant with DAPI (Invitrogen™, Carlsbad, CA, USA). The samples were observed by Fluoview^TM^ FV1000 confocal microscopy (Olympus, Tokyo, Japan).

### 2.6. Electron Microscopy

PR8, scPR8-RBD-M2 and scPR8-mCherry-M2 were concentrated by ultracentrifugation through 20% glycerol in PBS. Five microliters of each virus sample were negatively stained with 3% phosphotungstic acid solution (PTA) for 30 sec. The virus/PTA mixture was incubated on a carbon-coated copper grid (EMS) and incubated for 1 min. For immunogold labeling, the virus/PTA mixture was adsorbed onto a carbon-coated copper grid for 30 min followed by blocking with 1% bovine serum albumin (BSA) for 1 h. The grids were then incubated with rabbit anti-Spike RBD monoclonal antibodies (Sino Biological, Beijing, China) at 1:30 dilution in PBS for 1 h and rinsed six times with PBS, followed by incubation with goat anti-rabbit IgG antibodies (conjugated with 10-nm gold particles) at 1:100 dilution in PBS for 1 h. After washing, the samples were fixed for 5 min in 2% glutaraldehyde and negatively stained with 3% PTA. The grid was kept in a vacuum incubator before observing by transmission electron microscope (HITACHI H7700, Tokyo, Japan) at 80 kV.

### 2.7. Western Blot Analysis

Cells were collected and re-suspended in the Pierce™ mammalian cell lysis buffer (Thermo Scientific, Waltham, MA, USA). Protein samples were loaded onto polyacrylamide gel, and transferred to nitrocellulose membranes. Membranes were probed with rabbit anti-Spike RBD, anti-S (Sino Biological), anti-influenza A NP (FluA NP 4F1) (Southern Biotech), Mouse anti-2A (Novus, Littleton, CO, USA). Goat anti-rabbit IgG-HRP (KPL, MA, USA) and anti-mouse IgG-HRP antibodies were used as secondary antibodies for chemiluminescence detection by ChemiDoc™ XRS+ imager (Bio-Rad, Hercules, CA, USA).

### 2.8. Mice Vaccination via Prime-Boost Regimens and Sample Collection

To determine vaccine immunogenicity in the mouse model, 8-week-old female BALB/c mice were purchased from Nomura Siam International Co., Ltd. (Bangkok, Thailand). Mice were divided into 5 groups (*n* = 5/group) and received the vaccine using two alternative prime-boost (3-week interval) regimens including (i) intranasal-intranasal (IN-IN) and (ii) intranasal-intramuscular (IN-IM) routes. Mice were anesthetized with isoflurane before vaccination. For the first dose, mice were intranasally infected with scPR8-RBD-M2 or scPR8-mCherry-M2 (30 µL of 10^5^ TCID_50_/_mL_/mouse, passage 5). At 3 weeks after the first vaccination, mice were subsequently intranasally or intramuscularly boosted with the same dose of viruses. Throughout the immunization period, percentage of body weight loss and clinical signs of infection (hunching, ruffling of fur, malaise, or respiratory distress) were monitored. At 3 weeks after boosting, mice were sacrificed for sample collection (serum, trachea, lung and spleen). All mouse experiments were conducted following the guidelines of the Institutional Animal Care and Use Committee (IACUCs), National Center for Genetic Engineering and Biotechnology (BT-Animal 22/2563).

### 2.9. ELISA

The trachea and lung were homogenized in 400 µL PBS and centrifuged at 10,000× *g* for 5 min to collect the supernatants. Mouse sera and trachea/lung supernatants were subjected to ELISA to determine specific IgG and IgA, respectively, against SARS-CoV-2 spike. A flat-bottom 96-well ELISA plate (Corning Inc., Corning, NY, USA) was pre-coated with 5 µg/mL SARS-CoV-2 spike protein purified from HEK293T cells transfected with pCAGGS expressing soluble SARS-CoV-2 spike (a kind gift from Dr. Florian Krammer distributed via BEI resources). After blocking with 1% BSA (Sigma Aldrich, St. Louis, MO, USA), serially diluted mouse sera or trachea/lung supernatants were added into each well and incubated at room temperature for 1 h. Next, the plates were washed with PBST (0.1% Tween-20) and incubated with HRP-conjugated goat antibodies against mouse IgG (KPL) or mouse IgA (Abcam) for 1 h. After washing, the substrate 3,3′,5,5′-tetramethylbenzidine (Biolegend, San Diego, CA, USA) was added to the plates and the reaction was stopped by adding 1N H_2_SO_4_. The absorbance was measured by Synergy, HTX multi-mode reader at 450 nm (Bio Tek, Winooski, Vermont, USA). The flat-bottom 96-well ELISA plate coated with purified PR8 HA (Sino Biological, Beijing, China) was used to measure IgG specific to PR8 HA in mouse sera.

### 2.10. IFN-γ ELISPOT Assay

According to the manufacturer’s protocol, the assay was performed using an ImmunoSpot^®^ mouse IFN-γ ELISPOT kit (CTL, Shaker Heights, OH, USA). Briefly, 96-well ELISPOT plates were activated by adding 15 µL of 70% ethanol per well and washed 3 times with 1 x PBS. The plates were then coated with anti-murine IFN-*γ* antibodies diluted in murine IFN-*γ* capture solution at 4 °C overnight. Next, single-cell suspensions prepared from the spleens of vaccinated mice were added to the wells (3 × 10^5^ cells/well) and incubated with pools of overlapping 15-mer peptides spanning the entire length of SARS-CoV-2 spike protein (JPT Peptide Technologies, Berlin, Germany) or NP peptide 147–158 [18] (JPT Peptide Technologies, Berlin, Germany) at 37 °C, 5% CO_2_ for 24 h. After washing the plates with PBS and 0.05% Tween-PBS, biotinylated-labeled anti-mouse IFN-*γ* mAbs were added to each well and incubated at room temperature for 2 h. After additional washes, streptavidin-conjugated alkaline phosphatase was added and further incubated at room temperature for 30 min. Lastly, 5-Bromo-4-Chloro-3-Indolyl Phosphate (BCIP) solution was added to measure IFN-*γ*-producing T cell spots by an automated ELISPOT reader system and ImmunoSpot 3 software (Cellular Technology, Shaker Heights, USA). Results were expressed as the number of spot-forming cells (SFC) per 10^6^ input cells.

### 2.11. SARS-CoV-2 Pseudotyped Virus Neutralization Assay

Pseudotyped viruses (PV) with the firefly luciferase reporter displaying the full-length spike protein of various SARS-CoV-2 variants of concern (VOC), including Alpha, Beta, and Gamma were generated and titrated using methods as described previously [19]. Mouse sera were heat-inactivated at 56 °C for 30 min before use in the assay. The sera were incubated with 1 × 10^4^ relative light unit (RLU/mL) PV at 37 °C for 1 h and diluted in DMEM supplemented with 10% FBS before being transferred to each well of a tissue culture treated, white opaque 96-well microplate. HEK 293T cells expressing ACE2 and TMPRSS2 cells (1 × 10^4^ cells) were suspended in 50 µL DMEM supplemented with 10% FBS and then added into each well. The plate was incubated at 37 °C, 5% CO_2_ for 48 h. Supernatants were removed from each well before adding 25 µL of Bright-Glo^TM^ luciferase substrate (Promega, Madison, WI, USA). The luciferase signals (RLU/mL) correlating to the PV titers were measured using Synergy, HTX multi-mode reader (BioTek, Winooski, USA ) and normalized with the signal gained from no-serum control following the guideline described previously [19]. The half-maximal inhibitory dilution (ID50) of each serum tested or the half-maximal inhibitory concentration (IC50) of antibodies tested was calculated to determine the neutralization activity of each serum sample.

### 2.12. Statistical Analysis

All data were expressed as means ± standard error of means (SEM). The differences in mean values of between groups were analyzed by two-way ANOVA. *p* values < 0.05 were considered statistically significant. GraphPad Prism 9.0 (GraphPad Software, San Diego, CA, USA) was used for statistical analyses.

## 3. Results

### 3.1. Generation and Characterization of a Single Cycle PR8 Virus Harboring SARS-CoV-2 RBD

In order to generate the recombinant influenza gene that encodes the membrane-anchored receptor-binding domain (RBD) of SARS-CoV-2 spike presented on the influenza virus envelope, the RBD region fused with the transmembrane domain and cytoplasmic tail of PR8 HA (RBD-HAcyt) was constructed to replace the HA ORF of PR8 HA segment. The human tissue plasminogen activator signal sequence (tPA) was also included upstream of RBD-HAcyt to increase RBD-HAcyt protein expression and immunogenicity of the vaccine [20]. In fact, our first generation of scPR8-SARS-CoV-2 vaccine candidate (scPR8-RBD-HAcyt) (Supplementary Figure S1A) was constructed. However, the virus failed to maintain the chimeric RBD-HAcyt gene in its genome resulting in loss of RBD expression following viral propagation (Supplementary Figure S1B).

To maintain the stability of the inserted gene, a bi-cistronic protein expression cassette of RBD-HAcyt and M2 was generated by introducing 2A self-cleavage site between the RBD-HAcyt (or mCherry as the control) and M2 protein (Figure 1A). The chimeric gene cassette was named RBD-M2. We successfully generated a single cycle PR8 virus harboring SARS-CoV-2 RBD (scPR8-RBD-M2) and verified appropriate insertion of the RBD-M2 and mCherry-M2 ORF into the HA-encoding segment via RT-PCR (Supplementary Figure S2). We showed that RBD-M2 protein was expressed as shown by immunofluorescence assay (IFA) against α-RBD and α-Spike antibodies (Figure 1B) and Western blot analysis (Figure 1C) and maintained in the viral genome after multiple passages (Figure 1D). This is due to increased selective pressure to maintain the M gene as it was previously shown that influenza A virus M2 ion channel activity was essential for efficient replication of the virus [21]. To further verify the genetic stability of the RBD construct, cells infected with the virus at the eighth passage was subjected to Western blot to determine the RBD expression (Figure 1E). The virus at the eighth passage was also collected for RNA isolation and RT-PCR sent for direct nucleotide sequencing. No mutations were identified in the chimeric gene, indicating that the inserted sequences remain stable and error-free after several passages (data not shown). These results suggest that incorporating the functional M2 coding sequence adjacent to RBD-HAcyt compensates for a disrupted M2 gene in the M gene segment and, consequently, forces the virus to maintain the RBD-M2 gene cassette in its genome.

**Figure 2:**
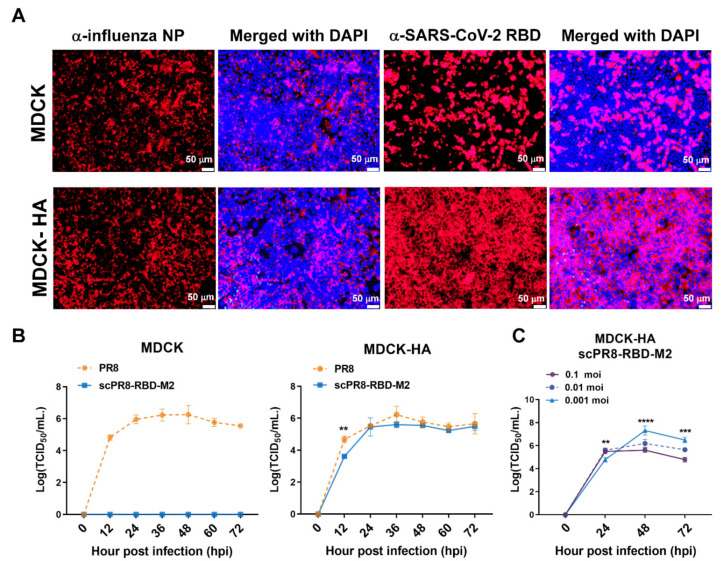
Replication of scPR8-RBD-M2 in MDCK cells. (**A**) MDCK and MDCK-HA cells were infected with scPR8-RBD-M2 and subjected to immunofluorescence assay. α-NP and -RBD antibodies were used to detect the presence of influenza NP and SARS-CoV-2 RBD in infected cells. Nuclear staining by DAPI dye was indicated. (**B**) scPR8-RBD-M2, scPR8-mCherry-M2 and parental PR8 viruses were inoculated in MDCK and MDCK-HA cells at MOI of 0.01. The viruses were then titrated at indicated time points. (**C**) MDCK-HA cells were infected with the scPR8-RBD-M2 virus at varied MOIs. Cell supernatants were collected to measure virus titers by TCID50 assay at indicated time points. Error bars represent the mean ± standard error of mean. ** *p* < 0.01, *** *p* < 0.001, **** *p* < 0.0001.

Immunofluorescence assay was also performed to confirm that an ectopic expression of HA was necessary for scPR8-RBD-M2 propagation. Wild-type MDCK and MDCK-HA cells were infected with scPR8-RBD-M2 at MOI of 0.1 for 24 h and probed with anti-NP and anti-RBD antibodies. The results showed that while MDCK and MDCK-HA are permissive to scPR8-RBD-M2 infection, only MDCK-HA could support the multi-cycle replication of scPR8-RBD-M2 (Figure 2A). The growth kinetics of scPR8-RBD-M2 and scPR8-mCherry-M2 were compared with the parental PR8 virus in both MDCK and MDCK-HA cells. MDCK cells were infected with the viruses at an MOI of 0.001. Cell supernatants were collected at indicated time points for virus titration in MDCK-HA. As expected, the growth of scPR8-RBD-M2 remarkably impaired in the wild-type MDCK cells (Figure 2B). In MDCK-HA cells, scPR8-RBD-M2 efficiently replicated, however, had lower titers than those of PR8 virus at 24 hpi (Figure 2B). We also showed that scPR8-RBD-M2, at an optimal MOI of 0.001, proliferated and reached the highest titer in MDCK-HA cells at 48 hpi (Figure 2C). Taken together, scPR8-RBD-M2 propagation requires the complement expression of HA in the producer cells, indicating that the virus cannot replicate multiple rounds in infected cells. Therefore, it should be safe for use in vivo where virtually all target cells do not naturally express the HA protein.

### 3.2. Detection of RBD on the Surface of Infected Cells and scPR8-RBD-M2 Virions

**Figure 3:**
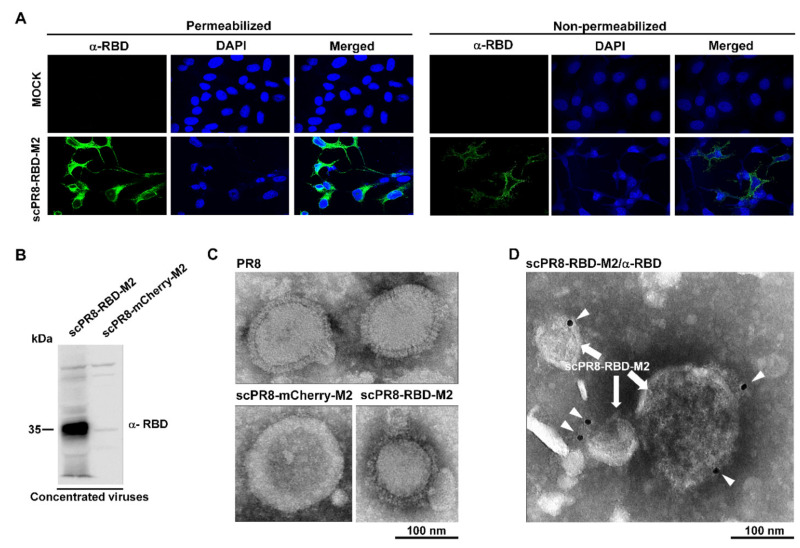
Presentation of RBD on the membrane of infected cells and viral particles. (**A**) Confocal microscopy displayed surface expression of a membrane anchored-RBD in infected MDCK-HA. MDCK-HA cells were infected with scPR8-RBD-M2 and prepared under non-permeabilized and permeabilized conditions for IFA. The cells were probed with rabbit anti-spike RBD antibody. Goat anti-rabbit IgG antibodies conjugated with Alexa flour 488 was used as secondary antibody. (**B**) scPR8-RBD-M2 and scPR8-mCherry-M2 viruses were centrifuged through 20% glycerol in PBS and re-suspended with PBS. The purified viruses were subjected to (**B**) Western blot analysis, (**C**) negative staining and (**D**) immuno-labeling determined by transmission electron micrograph (TEM). The virus particles were labelled with rabbit anti-RBD monoclonal antibodies conjugated to 10-nm gold particles.

We next sought to determine whether cells infected with scPR8-RBD-M2 expressed RBD on their surface. To this end, scPR8-RBD-M2-infected MDCK-HA cells were fixed and probed with anti-spike RBD antibodies at 48 hpi. RBD expressions were found localized on the surface of infected cells (Figure 3A). These results prompted us to speculate whether the RBD could also be found on the surface of newly assembled virions. To address this hypothesis, the morphology of scPR8-RBD-M2 was subjected to transmission electron microscopy analysis. MDCK-HA cells were infected with scPR8-RBD-M2 at an MOI of 0.1. At 72 hpi, the virus was collected and concentrated by ultracentrifugation. The presence of RBD was prominently detected in the purified virus (Figure 3B). Negative staining showed that scPR8-RBD-M2 and scPR8-mCherry-M2 particles exhibit spherical and lipid-bilayers similar to the WT PR8 virus (Figure 3C). To illustrate the localization of the membrane-anchored RBD on the virion particles, the virions were labeled with anti-RBD antibodies. While a few immunogold particles were observed on the scPR8-RBD-M2 virions (Figure 3D), none were seen to be co-localized with scPR8-mCherry-M2 particles (data not shown). The results indicate that a membrane-anchored RBD protein is present on the surface of nascent virions but might be masked by the abundant expression of the HA and NA proteins.

### 3.3. Prime-Boost Vaccination of scPR8-RBD-M2 Induced Both Humoral and Cell-Mediated Immune Responses to Influenza Virus Proteins and SARS-CoV-2 Spike

**Figure 4:**
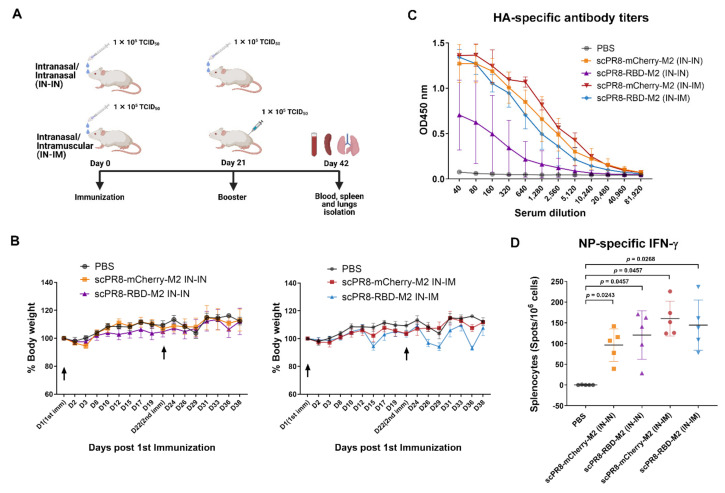
Immunization schedule and immune responses to influenza virus proteins. (**A**) Experimental design displaying vaccination schedule, routes, and sample collection. (**B**) Weight of individual mice was measured for the duration of study to monitor for any signs of disease. (**C**) HA specific antibody response measured by ELISA. (**D**) Splenocytes were stimulated with the conserved H2-K^d^-restricted NP peptide 147–158 [18]. Influenza A NP-specific T cells were quantified using ELISPOT assay. Error bars represent the mean ± standard error of mean. *p* values < 0.05 were considered statistically significant.

Five groups of female mice (five mice/group) were primed and boosted intranasally (IN-IN) or intranasally/intramuscularly (IN-IM) at a 3-week interval with 1 × 10^5^ TCID_50_ of scPR8-RBD-M2, scPR8-mCherry-M2 or PBS (Figure 4A). Mice were monitored post-immunization for signs of illness and weight loss. No clinical symptoms were observed in any group following IN-IN and IN-IM vaccination (Figure 4B). After the second dose, serum and tissue samples (trachea, lung and spleen) were collected for immunological assays. Despite lacking intrinsic HA gene, HA protein provided in trans by MDCK-HA was present on the surface of scPR8 viruses and sufficient to induce influenza-specific immune responses to HA (Figure 4C). To test whether scPR8-RBD-M2 viruses could induce cell-mediated immune responses to intrinsic influenza proteins, splenocytes isolated from vaccinated mice were stimulated with the conserved H2-K^d^-restricted NP peptide 147–158 [18]. The presence of NP-specific, IFN-γ producing T cells in the splenocyte samples were detected by ELISPOT assay. All tested viruses could induce strong NP-specific responses (Figure 4D), suggesting that our single-cycle viruses were able to infect mice and induce influenza virus-specific immune responses. It is also notable that while generating high serum HA-specific antibody levels (Figure 4C), none of the vaccinated mice displayed cell-mediated immune responses against HA (data not shown). As expected, HA-specific T cell responses are not remarkable, likely because the single-cycle viruses lack functional HA gene in their segmented genome.

**Figure 5:**
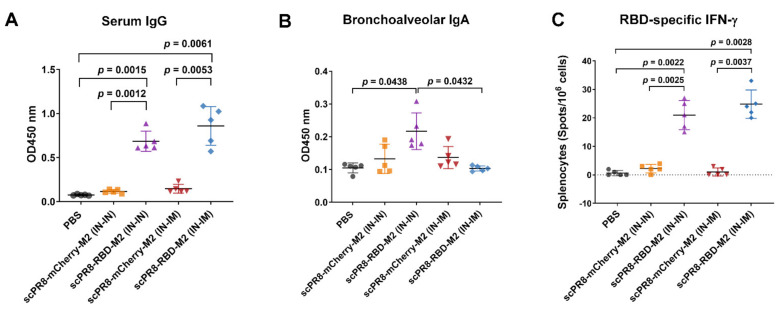
Cell-mediated and humoral immune responses elicited by scPR8-RBD-M2 in mice. Female BALB/c mice were intranasally (IN-IN) or intranasally primed followed by intramuscularly (IN-IM) boosted with 1 × 10^5^ TCID_50_/_mL_. PBS and scPR8-mCherry-M2 were used as controls. At 21 days post-second immunization, (**A**) titers of serum IgG (at 1:250 dilution) and (**B**) bronchoalveolar IgA antibodies (at 1:25 dilution) against SARS-CoV-2 RBD were determined by ELISA. (**C**) Splenocytes from vaccinated mice were stimulated with SARS-CoV-2 spike peptide pool. SARS-CoV-2 RBD specific T cells were quantified using ELISPOT assay. Error bars represent the mean ± standard error of mean. *p* values < 0.05 were considered statistically significant.

We next investigated whether scPR8-RBD-M2 could induce specific immune responses to the spike protein through different prime-boost strategies in mice. Serum ELISA shows that scPR8-RBD-M2 administered by both IN-IN and IN-IM induced robust anti-RBD antibody titers (Figure 5A). Interestingly, although both IN-IN and IN-IM triggered strong antibody responses, the serum antibody titers obtained from mice primed and boosted via IN-IM were slightly higher than those obtained from mice given the vaccine via the intranasal route alone (Figure 5A). Remarkably, scPR8-RBD-M2 enhanced induction of mucosal specific IgA antibody against SARS-CoV-2 RBD in mice that received the vaccine via prime-boost intranasal route compared to those given the virus through IN-IM route (Figure 5B). To further assess cell-mediated immune responses against spike-RBD, splenocyte single-cell suspension samples were stimulated with pools of overlapping 15-mer peptides spanning the entire length of the SARS-CoV-2 spike protein. The presence of IFN-γ specific T cells were quantified using ELISPOT assay. As shown in Figure 5C, scPR8-RBD-M2 induced a high level of SARS-CoV-2 RBD-specific IFN-γ ^+^ T cells in both IN-IN and IN-IM vaccination regimens, demonstrating that scPR8-RBD-M2 could induce strong SARS-CoV-2 RBD specific T cell responses.

### 3.4. Pseudotyped Viruses Bearing SARS-CoV-2 Spikes Were Potently Neutralized by Sera of scPR8-RBD-M2-Vaccinated Mice

**Figure 6:**
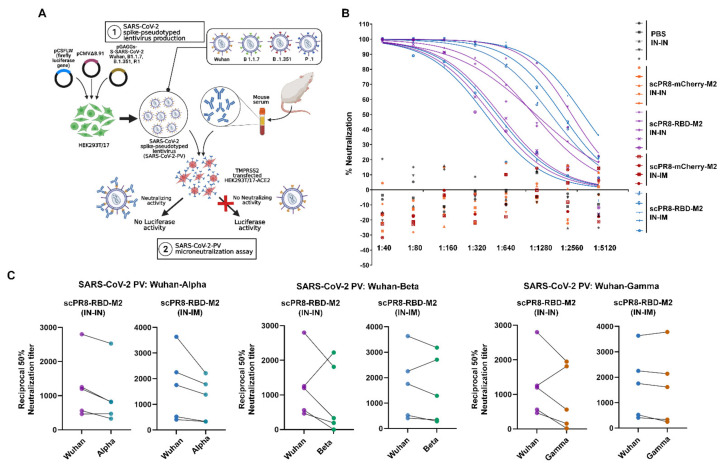
The scPR8-RBD-M2 induces neutralizing antibodies against SARS-CoV-2. (**A**) Schematic presentation of a spike pseudotyped virus (spike-PV) neutralization assay. SARS-CoV-2 spike protein sequences derived from Wuhan-HU-1, Alpha, Beta and Gamma variant were used for the generation spike-PV. (**B**) Percentage of neutralization activity of mice sera against Wuhan-PV. (**C**) Comparison between titers of neutralizing antibodies against spike-PV-Wuhan and spike-PV-variants (Alpha, Beta and Gamma) at 21 days post boost. Y-axis values show reciprocal dilution of the fifty-percent inhibitory concentration (IC_50_).

To determine whether serum anti-RBD antibodies confer a neutralizing activity, serum samples collected from immunized mice were subjected to spike pseudotyped virus (spike-PV) neutralization assay (Figure 6A). Both IN-IN and IN-IM immunization of scPR8-RBD-M2 induce a serum neutralizing antibody against pseudotyped virus displaying Wuhan-spike (Wuhan-PV) with IC_50_ values described (Figure 6B and Table 1). Notably, no neutralizing activity was observed against Wuhan-PV in mice inoculated with scPR8-mCherry-M2 or medium alone (Figure 6B). This result is in agreement with the notion that the majority of SARS-CoV-2 neutralizing humoral responses are accounted for by RBD-directed antibodies [22].

Within the past year since the beginning of COVID-19 pandemic, multiple SARS-CoV-2 variants have emerged and circulated globally. Several new variants appeared in the fall of 2020, most notably B.1.1.7 (Alpha variant), B.1.351 (Beta variant), and P.1 (Gamma variant). To assess whether RBD-specific antibodies obtained from scPR8-RBD-M2 immunized mice conferred neutralizing activity against new SARS-CoV-2 variants, pseudotyped viruses with Alpha, Beta or Gamma spike variant were generated. It is also noteworthy that spike-RBD specific antibodies induced by scPR8-RBD-M2 exhibit a cross-neutralization activity among spike proteins derived from distinct SARS-CoV-2 variants, despite a decreased of IC_50_ value shown (Figure 6C and Table 1). While PV neutralization titers slightly increased in a few sera samples (Table 1), we could not rule out the possibility that selective antibody clones induced by scPR8-RBD-M2 in those mice can recognize and neutralize spike proteins from heterologous strains.

## 4. Discussion

The application of viral vector systems for vaccine development against many infectious diseases has recently gained popularity. Influenza A viruses are the known causative agent of a highly contagious respiratory disease. Their preferred portal of entry through the respiratory mucosal epithelium makes the influenza A virus an attractive viral vector for the SARS-CoV-2 candidate vaccine development. A single cycle infectious influenza A virus (scIAV) as a vaccine platform for COVID-19 vaccine development was established in the present study. We report encouraging results of scIAV-vectored SARS-CoV-2 vaccine candidate as a safe vaccine and induce robust immune responses against the spike RBD of SARS-CoV-2. This advantage would potentially allow the use of single-cycle infectious influenza virus as a dual vaccine platform targeting both the seasonal flu as well as SARS-CoV-2.

The membrane-anchored form of the RBD domain of SARS-CoV-2 spike protein (Wuhan strain) was inserted into the HA gene segment of a laboratory-adapted influenza A/PR/8/34 (Figure 1A). Due to possible inherent genomic instability of the inserted foreign gene within the influenza genome, we engineered a bi-cistronic gene cassette for the simultaneous expression of the RBD and influenza M2 protein (RBD-M2) (Figure 1A), which significantly improved RBD-HAcyt stability in the influenza genome (Figure 2). A self-cleavage 2A peptide inserted between the coding sequence of RBD-HAcyt and M2 facilitates the co-expression of these two proteins allowing serial passaging and scalable production of the scPR8-RBD-M2 in MDCK-HA cells. While it was demonstrated that scPR8-RBD-M2 elicited spike-specific immune responses in mice, additional modification of scPR8-RBD-M2 to remove residual 2A motif retained in the C-terminal of the RBD-HAcyt protein could be applied [23]. Another consideration of utilizing influenza vectored is the size of the foreign gene to be packaged into the segmented genome of the influenza virus. While previous studies showed the feasibility of incorporating a foreign gene construct of approximately 1.5 kb into the influenza HA segment [24], PR8 HA protein provided in trans by MDCK-HA cells may lead to instability of the inserted gene maintenance. For this reason, a vaccine cocktail that contains scIAVs harboring mixed antigens/epitopes of interest could be generated to circumvent such limitation.

An ideal goal of vaccination is to induce humoral immune responses and cell-mediated immunity, leading to protective immunity upon subsequent exposure to relevant pathogens. While protective immunity against SARS-CoV-2 depends mainly on virus-specific antibody quantity and quality, recent studies in transgenic mouse models provided evidence that T-cell mediated clearance of the virus is also known to play essential roles in the complete resolution of the infection [25]. A cross-sectional study in convalescent individuals also correlated a robust T cell immunity and asymptomatic or mild COVID-19 [26]. Furthermore, it was shown that virus-specific T cell responses, particularly CD4^+^ T cells are associated with control of primary SARS-CoV-2 infection in humans [27]. Another study that showed a direct correlation between rapid induction of SARS-CoV-2-specific CD4+ T cells and mild clinical symptoms/accelerated viral clearance [27] recapitulated the importance of this subset of T cells in resolving SARS-CoV-2 infection. Our results demonstrated that the scPR8-RBD-M2 vaccine given to mice via two alternative prime-boost regimens could induce not only potent specific antibodies to SARS-CoV2 RBD but also spike-specific IFN-γ secreting T cells. While scPR8-RBD-M2 was able to induce the spike protein-specific T cells, further studies are required to demonstrate whether the presence of these T cell populations directly correlates with anti-viral activity. It is also of great interest to elucidate if spike-specific IFN-γ secreting T cells possess cytotoxic activity against SARS-CoV-2 infected cells. It is also important to emphasize that mice received scPR8-RBD-M2 via prime-boost intranasal route elicited local spike-specific IgA antibody production in trachea and lung samples.

By profiting from the already established cell culture-based system in influenza vaccine production, it is feasible to use a replication-deficient IAV harboring the highly immunogenic spike RBD gene as a candidate vaccine against two major respiratory viruses: IAV and SARS-CoV-2. Despite its potential as a promising dual-specific vaccine candidate, certain caveats still need to be addressed. Most importantly, while we validated that vaccinated mice display both the induction of spike-specific IFN-γ producing T cells and the neutralizing activity, we did not directly test if immune responses elicited by scPR8-RBD-M2 are protective against SARS-CoV-2. Additionally, as observed in the use of other vectored vaccine platforms [28,29,30], the issue concerning a pre-existing influenza-specific immunity could potentially affect the effectiveness of this vaccine. While pre-existing immunity against PR8 virus may no longer exist in the population nowadays, it will be beneficial to create MDCK cell line stably expressing HA from subtypes that are less prevalent in the human population. As a result, recombinant influenza viruses generated from such complementary cell lines could be used as subsequent booster doses. In addition to humoral immunity to the surface proteins including HA or NA, the cellular responses against the highly conserved influenza internal proteins such as NP or M1 could also potentially limit the efficacy of scIAV-based vaccines. Accordingly, the influenza virus strains to be used as virus vectors have to be chosen more carefully to avoid cross-reactive immune responses from pre-existing cell-mediated immunity. It is also worth mentioning that both humoral and cellular immune responses against the influenza virus vector could potentially have an impact on the annual seasonal influenza vaccination campaign. The selection of the future vector strains could thus be based on the official recommendation from the World Health Organization (WHO) to complement each annual seasonal influenza vaccination campaign. Additionally, despite a lack of replication competency, the scIAV based vaccine is a live virus that could confer undesired harmful side effects in vaccinated individuals. Further toxicity study in animal models is thus required to address this concern. Another important concern which needs to be addressed is the risk of the viral genome reassortment. While scPR8 virus contains six fully functional viral segments which can potentially reassort with those of natural influenza viruses in case of a simultaneous infection, it is less likely to produce newly emerged influenza strains with high virulence due to the low pathogenicity of PR8 in humans. In conclusion, the continuing threat of the ongoing SARS-CoV-2 pandemic and the emergence of new VOC make it clear that innovative vaccines are still needed. However, despite the caveats, using a scIAV-based vaccine platform provides the opportunity to simultaneously target two of the most life-threatening respiratory infections in the human population. The flu-based vector platform presents itself as a promising vaccine candidate to combat this pandemic with many advantages. These include a well-established reverse genetics system, being easily amendable to keep up with the changes among the SARS-CoV-2 variants, and existing infrastructure for the large-scale production of influenza virus-based vaccines.

## Figures and Tables

**Table 1:** Neutralization Titers (IC_50_) against pseudotyped viruses displaying each spike variant.

	IN-IN (Prime-Boost)			IN-IM (Prime-Boost)		
	Wuhan	Alpha Variant	Neutralization Activity (%) (Alpha/Wuhan)	Wuhan	Alpha Variant	Neutralization Activity (%) (Alpha/Wuhan)
#1	2801.50	2528.20	90.24	1754.7	1381.1	78.70
#2	1199.00	824.39	68.75	2253	1785.3	79.24
#3	1253.40	814.76	65.00	3633.8	2213.5	60.91
#4	561.35	328.49	58.51	522.44	333.29	63.79
#5	464.40	471.03	101.42	407.48	332.19	81.52
	**Wuhan**	**Beta** **Variant**	**Neutralization** **Activity (%)** **(Beta/Wuhan)**	**Wuhan**	**Beta** **Variant**	**Neutralization** **Activity (%)** **(Beta/Wuhan)**
#1	2801.50	1810.80	64.63	1754.7	1289.4	73.48
#2	1199.00	331.83	27.67	2253	2706.7	120.13
#3	1253.40	2226.50	177.63	3633.8	3181.6	87.55
#4	561.35	0.70	0.12	522.44	284.15	54.38
#5	464.40	190.63	41.04	407.48	343.41	84.27
	**Wuhan**	**Gamma Variant**	**Neutralization** **Activity (%)** **(Gamma/Wuhan)**	**Wuhan**	**Gamma Variant**	**Neutralization** **Activity (%)** **(Gamma/Wuhan)**
#1	2801.50	1949.00	69.56	1754.7	1613.4	91.94
#2	1199.00	561.39	46.82	2253	2138.3	94.90
#3	1253.40	1814.10	144.73	3633.8	3782.7	104.09
#4	561.35	18.52	3.29	522.44	248.49	47.56
#5	464.40	157.43	33.89	407.48	336.46	82.57

## Data Availability

The data presented in this study are contained within the article.

## Supplementary Materials

The following are available online at https://www.mdpi.com/article/10.3390/vaccines9080850/s1, Figure S1: Generation of scPR8 containing receptor-binding domain (RBD) of SARS-CoV-2 spike protein. Figure S2: Verification of RBD-M2 and mCherry-M2 insertion in the HA-encoding segment via RT-PCR.
