# Supplementary material for: A Single-Cycle Influenza A Virus-Based SARS-CoV-2 Vaccine Elicits Potent Immune Responses in a Mouse Model
Source: Vaccines (Basel). 2021 Aug 3;9(8):850. doi: 10.3390/vaccines9080850 (PMC8402467; doi:10.3390/vaccines9080850)
Supplement: Supplementary file 1 [file vaccines-09-00850-s001.zip › vaccines-1290269-supplementary.pdf]

vaccines

MDPI

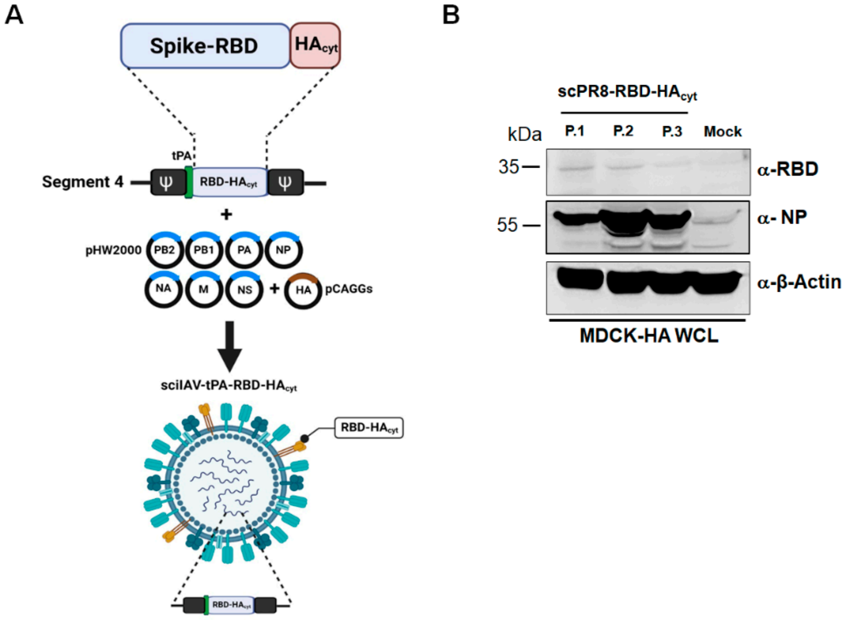


**Figure S1**. Generation of scPR8 containing receptor binding domain (RBD) of SARS-CoV-2 spike protein. (A) Schematic diagram of a reverse genetically generated scPR8-RBD-HAcyt. The RBD-HAcyt fusion gene was introduced in the segment 4 (HA) of A/PR/8/34 replacing most HA coding sequences. The packaging signal sequence (Ψ) at both 3′ and 5′ terminal ends are also depicted. (B) scPR8-RBD-HAcyt infected MDCK-HA were assessed for the RBD-HAcyt expression. Lysates of infected cells were subjected to Western blot analysis against $\alpha$-RBD, -NP and $-\beta$ actin antibodies. Mock infected MDCK-HA cells were used as a control.

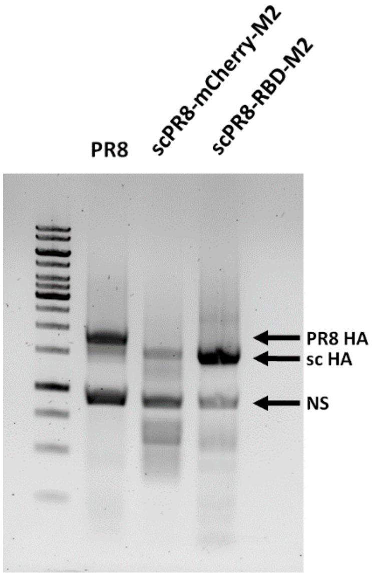


**Figure S2.** Verification of RBD-M2 and mCherry-M2 insertion in the HA-encoding segment via RT-PCR. Parental PR8, scPR8-mCherry-M2 and scPR8-RBD-M2 viruses were rescued and subjected to viral RNA isolation. RT-PCR reactions were performed using specific primers to HA and NS genes. PCR product sizes of HA gene derived from PR8, scPR8-mCherry-M2 and scPR8-RBD-M2 viruses are 1,741 bp, 1,071 bp and 1,203 bp, respectively. PCR product size of NS gene is 890 bp.
